# Ellagic Acid Attenuates Oxidative Stress and Improves Cardiovascular Function Following Myocardial Infarction in Ovariectomized Rats

**DOI:** 10.1111/jcmm.71262

**Published:** 2026-06-26

**Authors:** Laís Lopes Gonçalves, Ildernandes Vieira Alves, Erika Ferreira Nicoli, Deiviany Santana Santos Lima, Virginia Soares Lemos, A. Augusto Peluso, Patrick Wander Endlich, Simone Alves de Almeida, Glaucia Rodrigues de Abreu

**Affiliations:** ^1^ Department of Physiological Sciences, Health Sciences Center Federal University of Espírito Santo Vitória Brazil; ^2^ Mucuri School of Medicine, Graduate Program in Health Sciences Federal University of Jequitinhonha and Mucuri Valleys Teófilo Otoni Brazil; ^3^ Department of Physiology and Biophysics Federal University of Minas Gerais Belo Horizonte Brazil; ^4^ Faculty of Health and Medical Sciences University of Copenhagen Copenhagen Denmark

**Keywords:** cardiac fibrosis, cytokines, ellagic acid, nitric oxide, ovariectomy, oxidative stress

## Abstract

Oestrogen deficiency increases oxidative stress and reduces nitric oxide (NO) bioavailability, contributing to cardiac fibrosis following myocardial infarction (MI). This study investigated whether ellagic acid (EA), a natural antioxidant, attenuates post‐MI cardiac fibrosis in ovariectomized rats by reducing oxidative stress and pro‐inflammatory mediators. MI was induced by coronary artery ligation, and EA (30 mg/kg) was administered orally for 4 weeks. Hemodynamic parameters, collagen deposition, infarct size, matrix metalloproteinase‐8 (MMP‐8), NO, superoxide anion and inflammatory cytokines were assessed. MI resulted in ventricular dysfunction, increased collagen deposition and elevated levels of MMP‐8, interleukin‐6 (IL‐6) and superoxide anion. EA treatment improved hemodynamic function, reduced collagen deposition, decreased MMP‐8, IL‐6 and superoxide anion, and enhanced NO bioavailability. These findings demonstrate that EA mitigates cardiac fibrosis and ventricular dysfunction through antioxidant and anti‐inflammatory mechanisms, highlighting its potential as a cardioprotective compound in post‐MI conditions under oestrogen deficiency.

**Abbreviations**
a.u.: arbitrary units
BP: blood pressure
BSA: bovine serum albumin
CAPES: Coordenação de Aperfeiçoamento de Pessoal de Nível Superior
CEUA: Ethics Committee on Animal Use
cGMP: cyclic guanosine monoPhosphate
COX‐2: cyclooxygenase‐2
CVD: cardiovascular diseases
DAF‐2DA: 4,5‐diaminofluorescein diacetate
DBP: diastolic blood pressure
DHE: dihydroethidium
dP/dt+: maximum derivative of LV contraction
dP/dt−: maximum derivative of LV relaxation
EA: ellagic acid
ECM: extracellular matrix
ELISA: enzyme‐linked immunosorbent assay
eNOS: endothelial nitric oxide synthase
FAPES: Fundação de Amparo à Pesquisa do Espírito Santo
GTP: guanosine triphosphate
HR: heart rate
IL‐6: interleukin‐6
i.p.: intraperitoneal
LAD: left anterior descending
LV: left ventricular
LVEDP: left ventricular end‐diastolic pressure
LVSP: left ventricular systolic pressure
MAP: mean arterial pressure
MI: myocardial infarction
MMP‐8: matrix metalloprotease‐8
NIH: National Institutes of Health
NO: nitric oxide
OVX: ovariectomized
PFA: paraformaldehyde
PKG: protein kinase G
ROS: reactive oxygen species
SBP: systolic blood pressure
SEM: standard error of the mean
TNF‐α: tumour necrosis factor‐alpha

## 1 Introduction

Cardiovascular diseases (CVD) are the leading causes of death and morbidity worldwide [1]. Although women are less likely to suffer from CVD than men of the same age, the reduction in ovarian hormones, whether due to menopause or bilateral ovariectomy [2], reduces cardiovascular protection and increases the risk of developing CVD, including acute myocardial infarction (MI) [3, 4, 5].

After MI, cardiac tissue undergoes pathological remodelling, marked by reorganisation of the extracellular matrix (ECM) and a decline in cardiac function [6]. The extent of remodelling is influenced by the severity of the injury, inflammation levels and oxidative stress. In many cases, this can lead to excessive collagen deposition, resulting in cardiac fibrosis, which is a major contributor to heart failure. Key molecular mediators of this process include matrix metalloproteinase‐8 (MMP‐8), tumour necrosis factor‐alpha (TNF‐α) and interleukin‐6 (IL‐6) [7, 8, 9].

Ellagic acid (EA) is a naturally occurring polyphenol abundant in red fruits, nuts and vegetables, and is recognised for its potent antioxidant properties [10]. By mitigating the damaging effects of reactive oxygen species (ROS), EA is considered a promising therapeutic candidate for the treatment of cancer, liver fibrosis, neurological disorders and CVDs [11, 12, 13]. Recent studies have demonstrated the cardioprotective effects of EA in experimental models of cardiovascular disorders. EA improved endothelial function and reduced blood pressure in hypertensive rats, which was associated with enhanced vasodilatory responses and restoration of nitrate/nitrite levels, suggesting improved nitric oxide (NO) bioavailability and attenuation of oxidative stress [12, 14]. In addition to its antioxidant effects, EA exhibits anti‐inflammatory properties. Previous studies have demonstrated that EA reduces cyclooxygenase‐2 (COX‐2) levels in human aortic endothelial cells exposed to glucose overload [15] and decreases circulating TNF‐α levels in experimental models of metabolic dysfunction and diabetes [16, 17]. Moreover, EA attenuated pulmonary hypertension, ventricular hypertrophy, oxidative stress, caspase‐1 activation, IL‐1ß signalling and inflammatory cytokine levels in monocrotaline‐treated rats [14].

We have previously shown that EA improves diastolic dysfunction by reducing ROS levels in infarcted ovariectomized (OVX) rats [18]. However, the impact of EA on cardiac fibrosis and its contribution to cardiac recovery remains unknown.

We hypothesised that EA attenuates post‐myocardial infarction cardiac fibrosis in OVX rats by reducing superoxide anion and pro‐inflammatory cytokine levels, whilst simultaneously enhancing NO production. Therefore, this study aimed to investigate the potential cardioprotective effects of EA in cardiac injury and elucidate the involvement of MMP‐8, TNF‐α and IL‐6 as mediating mechanisms.

## 2 Methods

### 2.1 Animals

Female Wistar rats, 8 weeks old and weighing between 160 and 200 g, were obtained from the central animal facility of the Federal University of Espírito Santo (UFES). A total of 52 animals were used in this study. The animals were housed in collective cages with free access to water and food, controlled temperature (22°C–24°C) and light–dark cycles (12–12 h). All experimental protocols adhered to the National Institutes of Health (NIH) guidelines for the use and care of laboratory animals and were approved by the Ethics Committee on Animal Use (CEUA) of the Health Sciences Centre of the Federal University of Espírito Santo (UFES) (protocol number: 07/2022). The animals were randomly divided into three experimental groups: (1) OVX, (2) OVX + MI and (3) OVX + MI + EA. Due to mortality associated with myocardial infarction surgery, one animal was lost in each infarcted group during the experimental protocol period. Additionally, one animal from the OVX group died due to complications during a subset of hemodynamic analysis. Thus, for hemodynamic analyses, the final sample sizes were OVX (*n* = 6), OVX + MI (*n* = 5) and OVX + MI + EA (*n* = 5). For the analysis of the infarct area, three animals per group (*n* = 3) were used. For fluorescence analyses, the final sample sizes were OVX (*n* = 5), OVX + MI (*n* = 5) and OVX + MI + EA (*n* = 5). One sample from the OVX + MI group could not be processed for DHE analysis. Therefore, the final sample size for this analysis was *n* = 4. For collagen deposition, immunofluorescence and cytokine determination by ELISA, *n* = 4 animals per group were used according to tissue availability and methodological requirements of each assay. Smaller sample sizes were adopted for histological and molecular analyses because these assays present lower biological variability and require specific tissue processing, following the ethical principles of reduction in animal use.

### 2.2 Ovariectomy

Animals were anaesthetised via intraperitoneal (i.p.) injection of ketamine and xylazine (50 mg and 10 mg/kg, respectively). Bilateral lateral incisions were made to exteriorize and remove the ovaries. The skin was sutured, and an intramuscular injection of enrofloxacin (2.5%, 0.1 mL) was administered to prevent postoperative complications.

### 2.3 Myocardial Infarction

Seven days after bilateral ovariectomy, the animals were anaesthetised with i.p. injection of ketamine and xylazine (50 mg/kg and 10 mg/kg, respectively) and subjected to surgery to induce MI by permanent occlusion of the left anterior descending (LAD) coronary artery [19]. Briefly, a lateral thoracotomy was performed at the level of the left fourth intercostal space to expose the heart, which was gently exteriorized, and the LAD artery was ligated using a 6.0 nylon monofilament thread mounted on a non‐traumatic needle. After the occlusion, the chest was immediately closed and sutured. The OVX group was subjected a sham surgical procedure, which consisted of the application of all the steps previously described, except for the occlusion of the coronary artery.

### 2.4 Ellagic Acid Treatment

Ellagic acid (≥ 95% by HPLC; molecular weight = 302.19 g/mol; CAS No. 476‐66‐4; Sigma, St. Louis, Missouri, USA) was diluted in saline (0.9% NaCl) and administered orally once at a dose of 30 mg/kg/day for 28 consecutive days, beginning 24 h after MI induction [18]. The OVX and OVX + MI groups received an equivalent volume of 0.9% NaCl, used as vehicle control.

### 2.5 Hemodynamic Assessment

To evaluate blood pressure (BP) and left ventricular (LV) function, animals were anaesthetised with i.p. injection of ketamine and xylazine (50 mg/kg and 10 mg/kg, respectively) 48 h after the last day of EA treatment. A polyethylene catheter (PE50) filled with heparinized saline (50 U/mL—Sodium Heparin, São Paulo, SP, Brazil) was inserted into the left carotid artery and connected to a pressure transducer (FE221 Bridge Amp, AD Instruments, Australia) coupled to a data acquisition system (Powerlab 4/35, AD Instruments, Australia). The catheter was then carefully advanced into the left ventricle for direct measurement of heart rate (HR), systolic BP (SBP), diastolic BP (DBP), mean arterial pressure (MAP), left ventricular systolic pressure (LVSP) and left ventricular end‐diastolic pressure (LVEDP). The maximum derivatives of LV contraction and relaxation (dP/dt+ and dP/dt−, respectively) were also recorded.

### 2.6 Cardiac‐Tissue and Plasma Collection

Forty‐eight hours after the end of the EA treatment period, all animals were deeply anaesthetised with ketamine (80 mg/kg) and xylazine (10 mg/kg) and subsequently euthanized by decapitation for cardiac‐tissue collection. Plasma samples were collected only from a subset of animals designated for cytokine determination.

### 2.7 Collagen Deposition Analysis

Transverse myocardial tissue sections (5 μm thick) were prepared and stained with Picrosirius Red (#365548, Sigma‐Aldrich, St. Louis, Missouri, USA) to visualise collagen content. A total of 20 images per group were acquired using a bright field microscope (Olympus, BX43, Tokyo, Japan) equipped with a 40× objective. Total collagen deposition was quantified as the red‐staining area normalised to the yellow‐staining tissue area using Image Pro Plus 32 and ImageJ software.

### 2.8 Measurement of Infarct Area

The infarcted area was delineated by Picrosirius Red staining. Images were captured at 100× and 400× magnification with a digital camera (Evolution, Media Cybernetics Inc., Bethesda, Maryland, USA) mounted on an optical microscope (Eclipse 400, Nikon, Tokyo, Japan). Infarct size was quantified as the mean percentage of the infarcted perimeter relative to the total left ventricular perimeter.

### 2.9 Immunofluorescence

Tissue sections were fixed with 4% Paraformaldehyde (PFA) for 15 min and then washed with 1% bovine serum albumin (BSA) in phosphate‐buffered saline (PBS). Blocking was performed using 3% BSA in 1× PBS containing 0.1% Triton‐X for 30 min at room temperature. After another wash with 1% BSA, slides were incubated overnight at 4°C in a humid chamber with the primary anti‐MMP8 antibody (1:1000, Invitrogen, #PA5‐28246, California, USA) diluted in the blocking solution. The following day, slides were washed and incubated with the secondary anti‐rabbit antibody (1:300, #A‐21428, Invitrogen, California, USA) for 1 h at room temperature. Nuclei were counterstained with DAPI (Vector Laboratories, California, USA), and slides were mounted and sealed. A total of 20 images per group were captured using an Apotome (Zeiss, Oberkochen, Germany) microscope with a 20× objective. Fluorescence intensity was quantified and normalised by the analysed area.

### 2.10 In Situ Detection of NO and Superoxide Anion Production

The production of was assessed using 4,5‐diaminofluorescein diacetate (DAF‐2DA, Merck, Darmstadt, Germany), a fluorescent probe sensitive to NO. Superoxide anion levels were evaluated using the Dihydroethidium (DHE, Sigma‐Aldrich, St.Louis, Missouri, USA) fluorescent probe. Heart cross‐sections were embedded in a freezing medium (Tissue‐Tek O.C.T. Compound, Sakura, Japan) and sectioned into 10 μm slices using a cryostat (Leica, CM1850, Wetzlar, Germany). Slides were incubated with 1× PBS at 37°C in a humidified, light‐protected chamber for 15 min, followed by incubation with either DAF‐2DA (8 μM) or DHE (10 μM) solution for 30 min. Slides were then fixed with 4% PFA for 5 min, mounted and sealed using Fluoromount (#00498502, Invitrogen, Massachusetts, USA) as the mounting medium. For image acquisition, 20 fields per group were captured using a fluorescence microscope (Apotome, Zeiss, Oberkochen, Germany) with a 40× objective. Fluorescence intensity was quantified using ImageJ software (https://imagej.net/ij/) to the normalised area. Results were expressed as relative fluorescence intensity in arbitrary units (a.u.).

### 2.11 Determination of Cytokines by ELISA

IL‐6 (#550319, BD Biosciences, California, USA) and TNF‐ɑ (#558535, BD Biosciences, California, USA) levels were quantified using a sandwich enzyme‐linked immunosorbent assay (ELISA). Blood samples were collected and centrifuged at 16,000×*g* for 20 min, and the plasma was collected for analysis. In addition, 50 mg of heart tissue was weighed, homogenised in cytokine extraction buffer and centrifuged to obtain the supernatant for analysis. Samples were prepared according to the manufacturer's instructions and analysed using a microplate reader (Agilent Biotek Epoch, Vermont, USA) at 492 nm. According to the manufacturer's specifications, the assay has an analytical sensitivity of 15 pg/mL and a dynamic detection range of 31.3 to 2000 pg/mL for TNF‐α, with intra‐assay and inter‐assay coefficients of variation (CVs) < 10% and < 12%, respectively. For the IL‐6 assay, the lower limit of quantification is 156 pg/mL, with a range extending up to 10,000 pg/mL and no significant cross‐reactivity with other cytokines (e.g., IL‐1, IL‐2, IL‐10 and TNF‐α) has been reported. The typical intra‐ and inter‐assay CVs for ELISA assays were < 10% and < 12%, respectively.

### 2.12 Statistical Analysis

Statistical analyses were performed using GraphPad Prism version 8.0 software. One‐way ANOVA followed by Tukey's multiple comparisons post hoc test was used to compare differences amongst experimental groups. Data are presented as mean ± standard error of the mean (SEM), and values of *p* ≤ 0.05 were considered statistically significant.

## 3 Results

### 3.1 Ellagic Acid Reduces Infarct Size and Improves Hemodynamic Parameters

**FIGURE 1:**
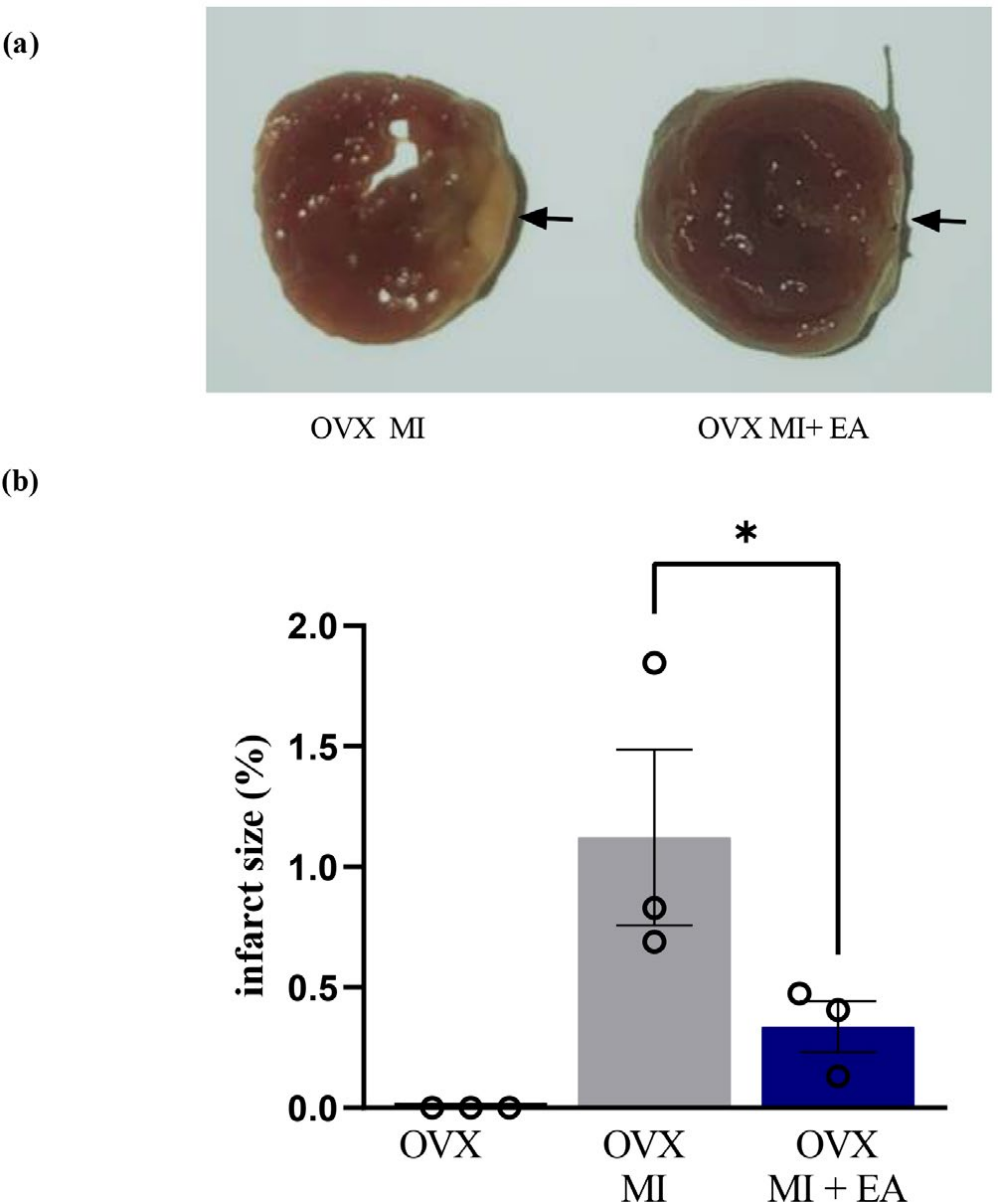
Effect of oral EA administration on myocardial infarct size in ovariectomized rats. (a) Representative images showing acute myocardial infarction (MI) size after coronary artery ligation in ovariectomized (OVX) rats with or without EA administration. (b) Quantification of infarct size shows that oral EA administration significantly reduced myocardial damage. *N* = 3 per group. Data are presented as mean ± SEM. *p < 0.05 by One‐way ANOVA followed by Tukey's post hoc test.

Data on morphometric parameters and 17β‐estradiol are presented in the Table S1 (Effects of ellagic acid treatment in ovariectomized rats subjected to myocardial infarction on body mass, dry uterus weight, plasma 17β‐estradiol and ventricular weight). After coronary artery ligation in OVX rats, EA was administered orally for 4 weeks to evaluate its therapeutic potential in MI. To assess the extent of cardiac damage, infarct size was first analysed. As shown in Figure 1, coronary artery ligation surgery successfully induced MI, and EA treatment significantly reduced the infarcted area compared to untreated MI controls (Figure 1a,b).

**FIGURE 2:**
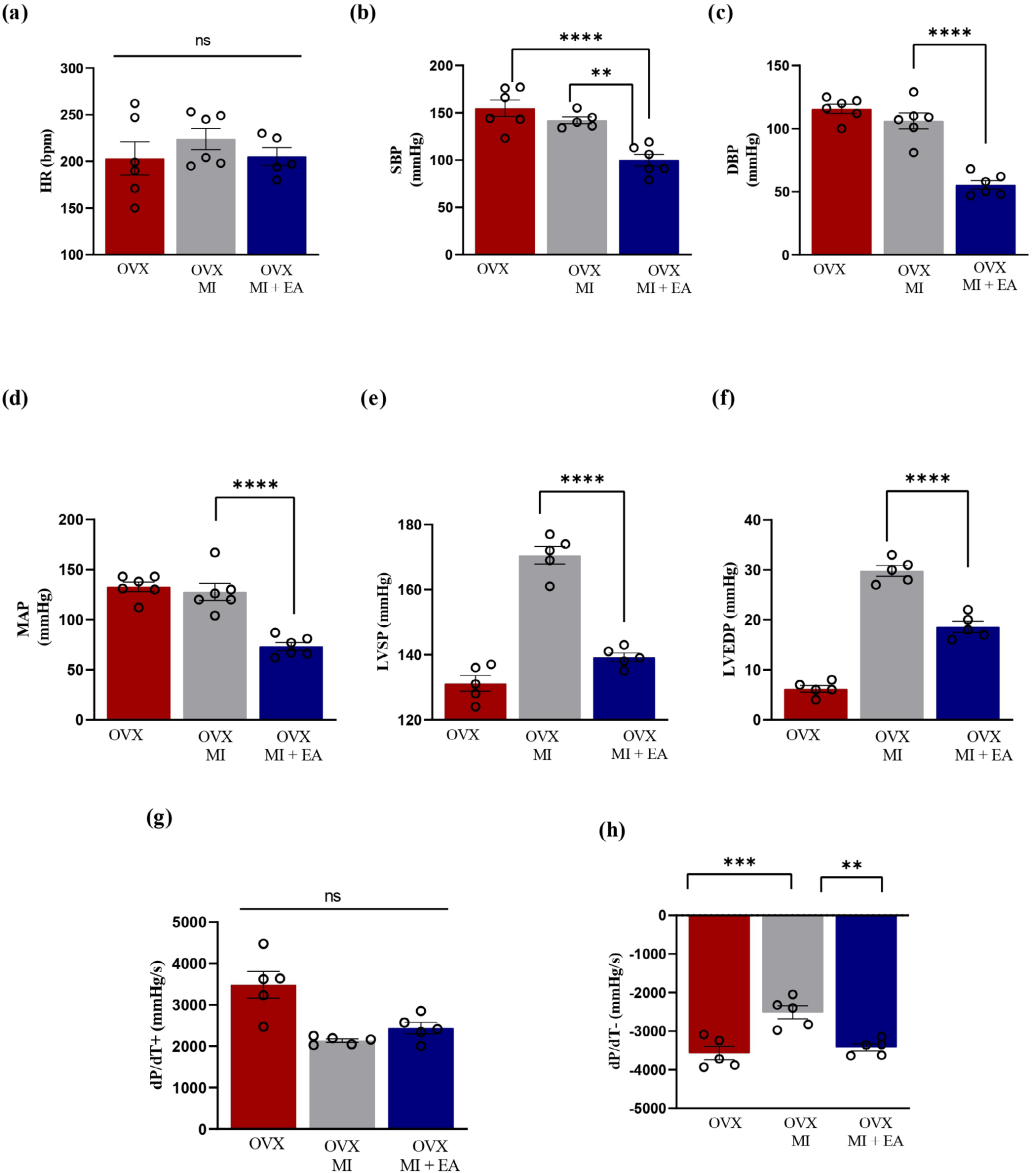
Effect of myocardial infarction and ellagic acid treatment on cardiovascular parameters in ovariectomized rats. Bar graphs show the effect of MI and EA treatment on (a) heart rate (HR), (b) systolic blood pressure (SBP), (c) diastolic blood pressure (DBP), (d) mean arterial pressure (MAP), (e) left ventricle systolic pressure (LVSP), (f) left ventricular end‐diastolic pressure (LVEDP), (g) the maximum rate of increase (dP/dt+), and (h) reduction (dP/dt‐) of left ventricular pressure in ovariectomized rats. *N* = 5–6 per group. Data are presented as mean ± SEM. *p < 0.05, **p < 0.01, ***p < 0.001, ****p < 0.0001 by one‐way ANOVA followed by Tukey's post hoc test.

Following MI induction, we investigated the cardioprotective effects of EA administration. As shown in Figure 2a–d, MI alone did not alter HR, SBP, DBP or MAP. However, EA treatment in MI rats resulted in a significant hypotensive effect, as evidenced by reductions in SBP, DBP and MAP. Furthermore, MI induced left ventricular dysfunction, characterised by increased left ventricular systolic pressure (LVSP) (Figure 2e), left ventricular end‐diastolic pressure (LVEDP) (Figure 2f), along with decreased rates of ventricular contraction (dP/dt+) (Figure 2g) and relaxation (dP/dt−) (Figure 2h). In line with this, EA administration effectively normalised LVSP and dP/dt− and reduced LVEDP in MI rats (Figure 2e–h).

### 3.2 Ellagic Acid Attenuates Collagen Deposition and Regulates Matrix Metalloproteinase 8 (MMP‐8) Levels in Infarcted Ovariectomized Rats

**FIGURE 3:**
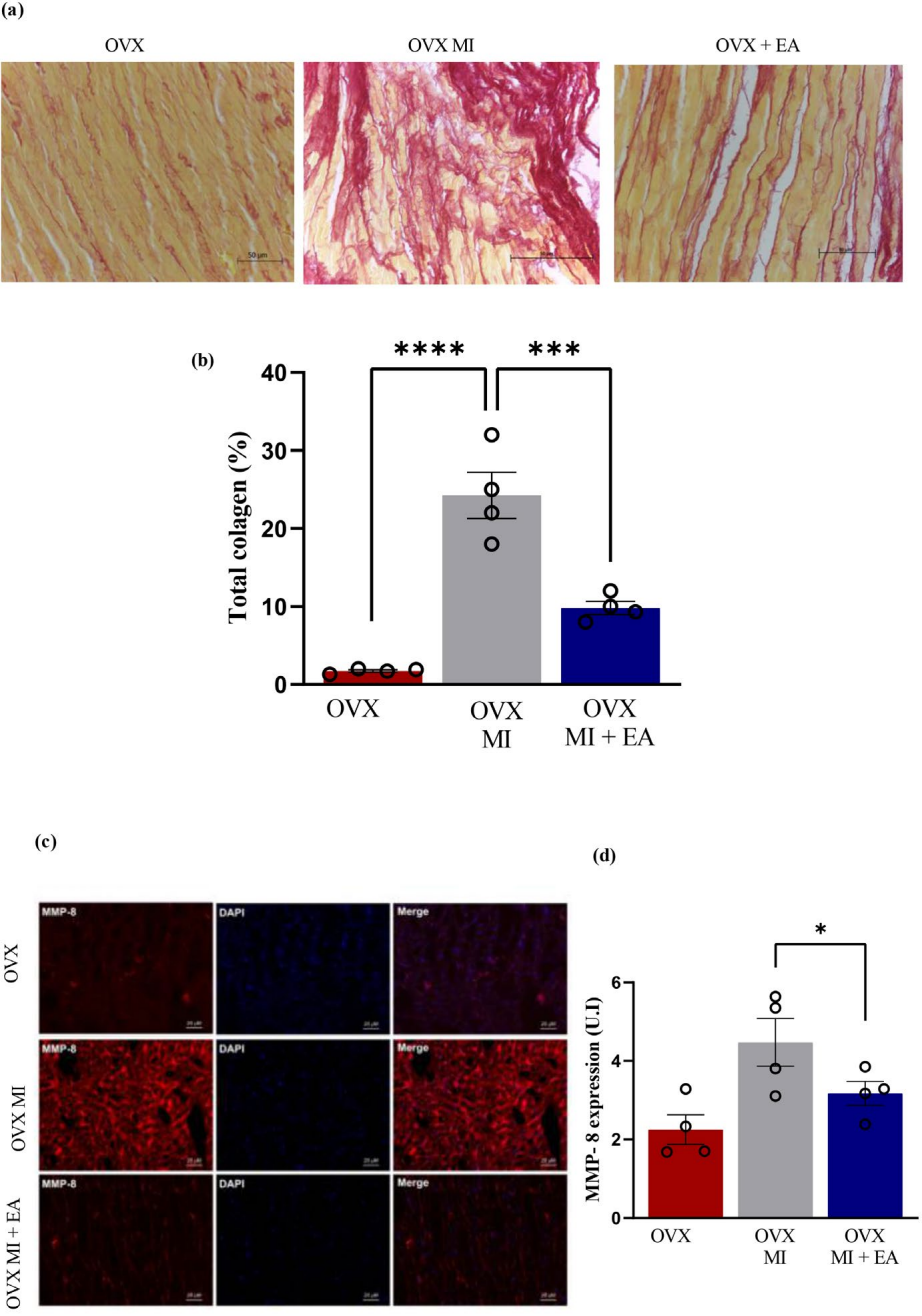
Effect of myocardial infarction and ellagic acid treatment on collagen deposition and MMP‐8 levels in ovariectomized rats. (a) Representative Picrosirius‐stained images (red), showing total collagen deposition in heart tissue (upper panel), 40× objective (b) quantification of total collagen deposition presented as a percentage of stained area, (c) representative immunofluorescence images showing MMP‐8 levels (red) in heart tissue. Nuclear staining with DAPI (blue) is shown in the middle panel, and merged images are presented on the right. 40× objective (d) quantification of MMP‐8 expression in the hearts of ovariectomized rats with MI, with or without EA treatment. *N* = 3–4 per group. Data are presented as mean ± SEM. Statistical analysis was performed using one‐way ANOVA followed by Tukey's post hoc test. * *p* < 0.05, ***p < 0.001, ****p < 0.0001.

To evaluate whether EA modulates this process, we assessed collagen deposition in infarcted hearts. As shown in Figure 3a,b, MI significantly increased total collagen deposition, whilst EA treatment markedly attenuated this fibrotic response. Similarly, MI induced a significant increase in MMP‐8 expression (Figure 3c,d), which was reduced following EA administration.

### 3.3 Ellagic Acid Increases NO Production and Reduces Superoxide Anion Generation in the Heart

**FIGURE 4:**
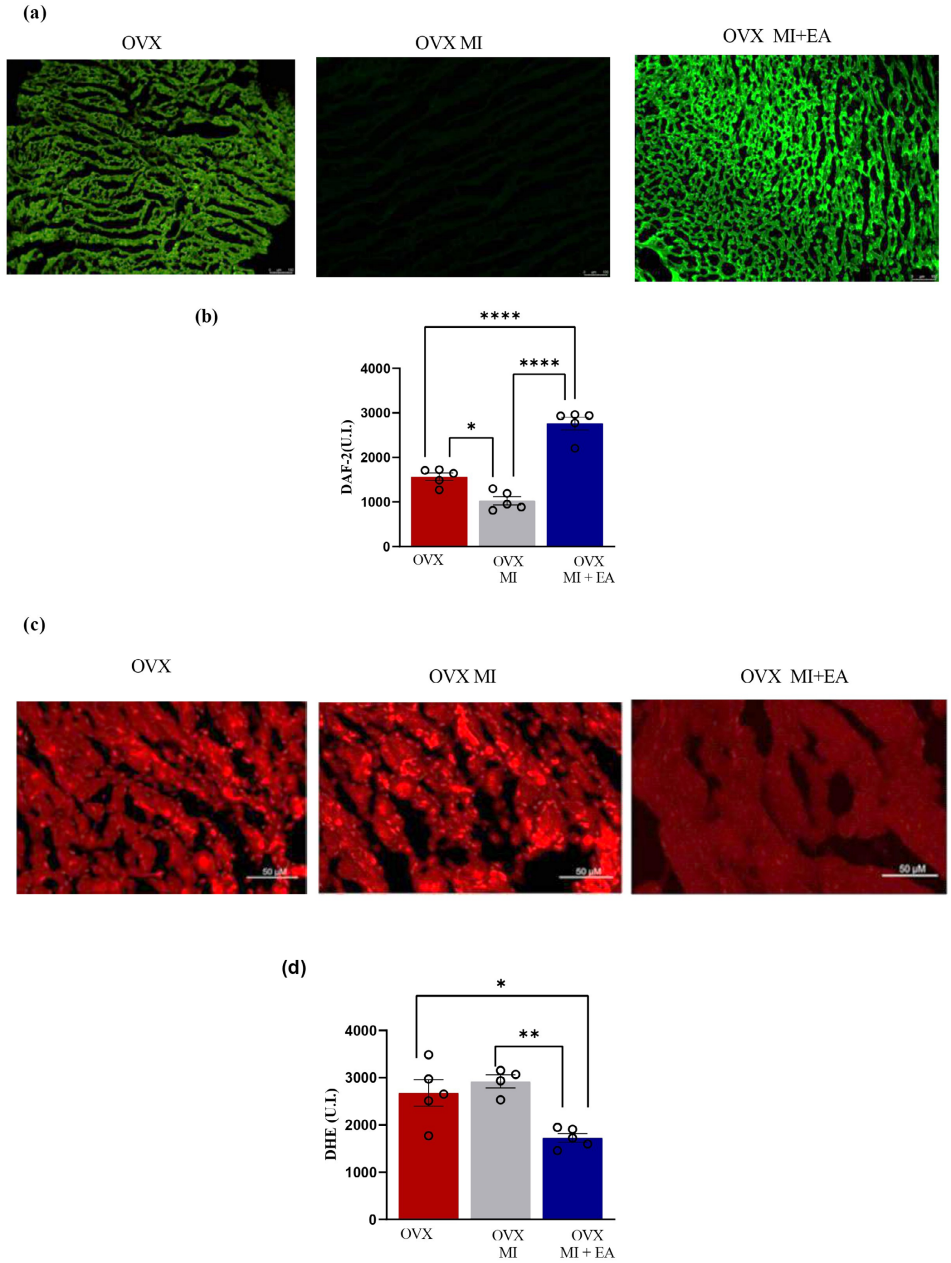
Effect of myocardial infarction and ellagic acid treatment on the nitric oxide (NO) and superoxide anion production in ovariectomized rats. (a) Representative fluorescence images showing NO levels in heart tissue using DAF‐2 staining, 20× objective (b) quantification of NO production based on DAF‐2 fluorescence intensity, (c) representative fluorescence images showing superoxide anion levels using DHE staining, 20× objective (d). Quantification of superoxide anion production based on DHE fluorescence intensity. *N* = 5 per group. Data are presented as mean ± SEM. Statistical analysis was performed using one‐way ANOVA followed by Tukey's post hoc test. *p < 0.05, **p < 0.01, ***p < 0.001, ****p < 0.0001.

To assess the effects of EA on NO availability, NO levels were measured in cardiac tissue. As shown in (Figure 4a,b), MI significantly reduced NO production, whereas EA treatment restored NO levels. Consistent with this, our results demonstrated elevated superoxide anion levels in the hearts of MI animals. Interestingly, EA treatment significantly reduced superoxide anion levels in OVX + MI rats, even below those observed in the OVX group alone (Figure 4c,d).

### 3.4 Ellagic Acid Reduces Cardiac IL‐6 Production After MI

**FIGURE 5:**
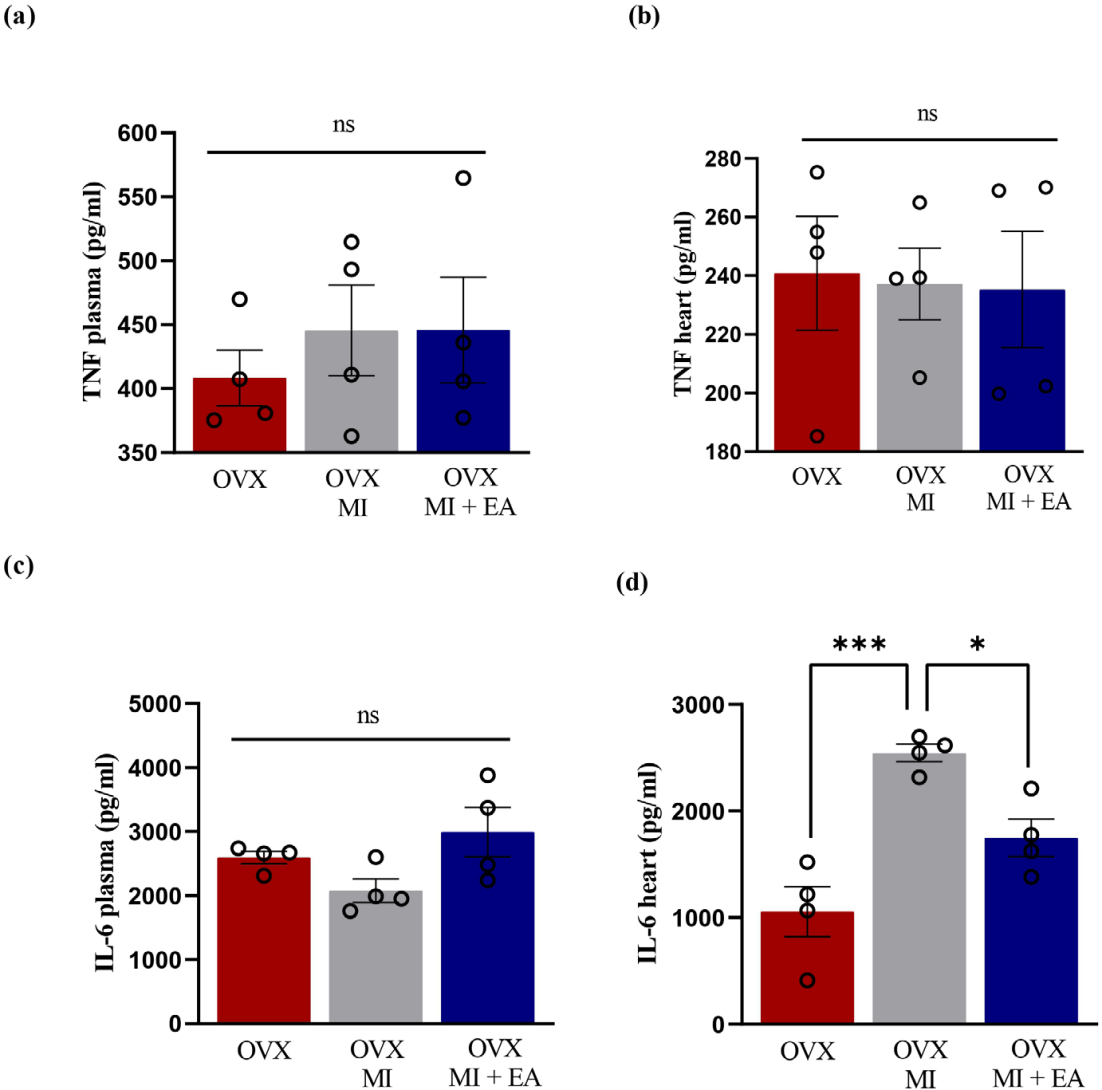
Effect of myocardial infarction and ellagic acid treatment on IL‐6 and TNF‐α levels in ovariectomized rats. Concentrations of TNF‐α (a, b) and IL‐6 (c, d) in plasma and heart, respectively, were measured using ELISA. *N* = 4 per group. Data are expressed as mean ± SEM. Statistical analysis was performed using one‐way ANOVA followed by Tukey's post hoc test. *p < 0.05, ***p < 0.001.

To investigate whether EA modulates this response, we evaluated IL‐6 and TNF‐α levels in both plasma and cardiac tissue after MI induction. TNF‐α levels remained unchanged in both compartments across the experimental groups (Figure 5a,b). However, a significant increase in IL‐6 levels was observed specifically in cardiac tissue after MI (Figure 5c,d), and EA treatment attenuated this increase.

## 4 Discussion

This study demonstrated that EA effectively attenuated the pathophysiological damage induced by MI in OVX rats. These cardioprotective effects were evidenced by a reduction of infarct size, improvements in hemodynamic parameters, decreased collagen deposition, which can suggest a reduction in cardiac fibrosis [7] and anti‐inflammatory and antioxidant mechanisms. A major therapeutic goal in MI management is to minimise infarct size, thereby limiting adverse hemodynamic effects and pathological cardiac remodelling [20, 21]. Women with reduced ovarian hormone levels, such as those in menopause or after surgical ovariectomy, experience worse post‐MI outcomes, including increased mortality and more severe clinical symptoms [22]. In this context, our results demonstrated that in OVX rats subjected to MI induction, EA treatment significantly reduced the infarct size and also improved ventricular function.

Collagen deposition is a critical component of cardiac remodelling following injury [23, 24, 25]. Excessive collagen accumulation contributes to cardiac fibrosis and ventricular dysfunction, potentially leading to heart failure [8, 26, 27]. To elucidate the mechanisms underlying the cardioprotective effects of EA, we examined its impact on MI‐induced collagen deposition. Previous studies have shown that EA reduces levels of profibrotic proteins and collagen synthesis in human cardiac fibroblasts [28] and also exerts an anti‐fibrotic effect on the liver [13]. Consistent with these findings, we report here for the first time that EA administration attenuates cardiac collagen deposition in OVX rats after MI induction, highlighting its direct anti‐fibrotic potential in the heart.

The degradation of ECM is primarily regulated by MMPs [29, 30]. Amongst them, MMP‐8 is particularly relevant due to its ability to cleave collagen types I and III, which are major components of cardiac tissue [31]. Notably, elevated MMP‐8 levels have been positively correlated with ventricular dysfunction after MI [32]. We observed a significant increase in MMP‐8 expression in the hearts of infarcted OVX rats, which was attenuated by EA administration. This finding aligns with a previous study demonstrating that EA can inhibit MMP expression in endothelial cells [33]. Thus, it is reasonable to hypothesise that EA modulates the MMP‐8 levels, contributing to a reduction in collagen deposition and thereby mitigating cardiac fibrosis in MI.

Increased levels of pro‐inflammatory cytokines are known to exacerbate myocardial tissue damage, impair cardiac function and promote progression of cardiac fibrosis [34, 35, 36]. Amongst these cytokines, IL‐6 has been strongly associated with the severity of cardiac injury [32]. Supporting its pathological relevance, a recent randomised, double‐blind clinical trial demonstrated that IL‐6 receptor blockade significantly reduced cardiac damage in patients with MI [37]. In agreement with this, our study showed that IL‐6 levels were significantly elevated in the hearts of OVX rats following MI, and this increase was markedly attenuated by EA administration. These results suggest that EA may exert cardioprotective effects, at least in part, by modulating the inflammatory response through IL‐6 regulation.

Inflammation‐associated genes are activated when IL‐6 binds to the IL‐6 receptor. The activation triggers a cascade of inflammatory responses that promote the proliferation of cardiac fibroblasts and the production of the ECM components [11, 18, 28]. The resulting ECM accumulation leads to myocardial stiffness, which impairs ventricular filling and ejection [38, 39]. To compensate, the heart increases its workload, resulting in disorganised cardiomyocyte hypertrophy [40], which further compromises contractile function. Additionally, IL‐6 disrupts cardiomyocyte homeostasis by downregulating the expression of SERCA2a, a critical protein for calcium reuptake into the sarcoplasmic reticulum, thus impairing cardiac relaxation. It also reduces the expression of ryanodine receptors, leading to diminished calcium‐induced calcium release and contractile force [41]. In this study, we found that EA administration reduced cardiac IL‐6 levels of infarcted OVX rats. These findings suggest that the anti‐inflammatory properties of EA may be the initiating mechanism by which it limits myocardial injury, mitigates cardiac fibrosis and improves hemodynamic function.

Several studies have demonstrated that oxidative stress regulates collagen synthesis, metalloproteinase activity and contributes to the progression of cardiac fibrosis [42, 43, 44]. Conversely, EA is a potent antioxidant compound with protective cardiovascular properties [18, 28, 45]. In the present study, we observed that MI increased superoxide anion generation in the cardiac tissue of OVX rats. Treatment with EA significantly reduced superoxide anion levels. This reduction in oxidative stress likely contributes to the antifibrotic effect observed, as evidenced by the decrease in total collagen deposition following EA administration. In addition to its antioxidant effects, several intracellular signalling pathways modulate cardiomyocyte contraction and relaxation, many of which are dependent on calcium fluxes within the cytoplasm, mitochondria and ECM [46].

A key regulator of cardiovascular function is NO, which has also been associated with the development of cardiac fibrosis [47, 48]. Endothelial nitric oxide synthase (eNOS) derived NO activates soluble guanylate cyclase, converting Guanosine Triphosphate (GTP) into cyclic Guanosine MonoPhosphate (cGMP), which then activates protein kinase G (PKG). PKG phosphorylates calcium channels in the sarcoplasmic reticulum, such as phospholamban. This enhances SERCA2a activity during cardiac relaxation, moving calcium from the cytoplasm back into the sarcoplasmic reticulum, lowering cytoplasmic calcium levels. As a result, actin and myosin interact less effectively, reducing cardiomyocyte contraction strength [38, 40, 41, 46]. Thus, NO plays a key role in improving ventricular relaxation, enhancing myocardial distensibility and mitigating pathological remodelling of the heart [18, 49, 50]. Taken together, our findings suggest that EA enhances NO production in cardiac tissue, contributing not only to its vasodilatory and anti‐inflammatory effects but also to its capacity to reduce myocardial stiffness and prevent structural damage following MI.

## 5 Conclusion

In infarcted OVX rats, EA significantly reduced cardiac lesion size, improved hemodynamic parameters, decreased collagen deposition, enhanced nitric oxide (NO) production and exerted both anti‐inflammatory and antioxidant effects. These findings indicate that EA may serve as a promising therapeutic agent for mitigating cardiac fibrosis and ventricular dysfunction following myocardial infarction, primarily through modulation of ECM remodelling, hemodynamic stabilisation and reduction of oxidative and inflammatory stress contributing to cardiac regeneration.

Nevertheless, further studies are warranted to strengthen the translational relevance of these findings. Future investigations should focus on elucidating the molecular mechanisms underlying EA‐mediated cardioprotection, including its effects on specific signalling pathways involved in fibrosis and oxidative stress. In addition, dose–response studies, long‐term evaluations and validation in other preclinical models are needed. Ultimately, clinical studies will be essential to determine the safety, efficacy and therapeutic applicability of EA in oestrogen‐deficient populations with myocardial infarction.

## Author Contributions

**Laís Lopes Gonçalves:** conceptualization, methodology, investigation, writing – original draft. **Erika Ferreira Nicoli:** investigation, formal analysis. **Simone Alves de Almeida:** supervision, methodology, data curation, formal analysis, writing – original draft. **A. Augusto Peluso:** conceptualization, writing – original draft. **Glaucia Rodrigues de Abreu:** supervision, project administration, funding acquisition, writing – original draft. **Patrick Wander Endlich:** conceptualization. **Ildernandes Vieira Alves:** investigation, formal analysis. **Virginia Soares Lemos:** data curation, formal analysis, writing – original draft. **Deiviany Santana Santos Lima:** data curation, writing – original draft.

## Funding

National Council for Scientific and Technological Development (CNPq—grant number 160408/2021‐0); Research and Innovation Support Foundation of Espírito Santo (FAPES—grant number 301/2021).

## Ethics Statement

All experimental protocols adhered to the National Institutes of Health (NIH) guidelines for the use and care of laboratory animals and were approved by the Ethics Committee on Animal Use (CEUA) of the Health Sciences Centre of the Federal University of Espírito Santo (UFES) (07/2022).

## Conflicts of Interest

The authors declare no conflicts of interest.

## Supporting information


**Table S1:** Effects of ellagic acid treatment in ovariectomized rats subjected to myocardial infarction on body mass, dry uterus weight, plasma 17β‐estradiol and ventricular weight.

## Data Availability

The data that support the findings of this study are openly available in Zenodo at https://doi.org/10.5281/zenodo.17557742, reference number https://doi.org/10.5281/zenodo.17557742.
