# Supplementary material for: Ellagic Acid Attenuates Oxidative Stress and Improves Cardiovascular Function Following Myocardial Infarction in Ovariectomized Rats
Source: J Cell Mol Med. 2026 Jun 26;30(12):e71262. doi: 10.1111/jcmm.71262 (PMC13309391; doi:10.1111/jcmm.71262)
Supplement: Supplementary file 1 — Table S1: Effects of ellagic acid treatment in ovariectomized rats subjected to myocardial infarction on body mass, dry uterus weight, plasma 17β‐estradiol and ventricular weight. [file JCMM-30-e71262-s001.docx]

# SUPPLEMENTARY MATERIAL

Female intact Wistar rats (not submitted to any surgical procedure or treatment) at 8 weeks of age were kept under the same controlled conditions as the animals in the experimental groups. During the 28 days of treatment of the other groups (vehicle or ellagic acid), these animals were also only handled by the researchers for the same period and were sacrificed at 13 weeks of age, in order to collect data on body mass, uterine weight, plasma 17β -estradiol (measured by the RTFI01469 Kit, Assay Genie Lt, Dublin, Ireland) and ventricular weight (Table 1). These data together demonstrate the hypoestrogenic condition of the ovariectomized rats.

**Table 1.** Effects of ellagic acid treatment in ovariectomized rats subjected to myocardial infarction on body mass, dry uterus weight, plasma 17β-estradiol, and ventricular weight.

|  | Intact (n=6) | OVX (n=6) | OVX MI(n=6) | OVX MI+EA(n=6) |
| --- | --- | --- | --- | --- |
| Initial Body Mass (g) | 186.4±3.4 | 168.4±8.1 | 161.6±5.3 | 161.5±8.5 |
| Final Body Mass (g) | 235.6±5.6 | 267.2±5.3 | 260±3.8 | 237.2±8.4 |
| Body Mass Gain (Δ%) | 26.98±4.9 | 73.2±14.7* | 61.26±4* | 44.5±6 |
| Dry Uterus Weight (g) | 0.88±0.07 | 0.09±0.03*** | 0.11±0.009*** | 0.15±0.02*** |
| Plasma 17β-estradiol  (pg/mL) | 99.4±4.3 | 9.1±0.2*** | 8.1±0.1*** | 7.8±0.4*** |
| Ventricula r Weight (g) | 0.67±0.01 | 0.99±0.14 | 1.81±0.05***, ###, | 1.03±0.1+++ |

Va lues are expressed as means ± SEM. **p<0.05* vs. Intact female Wistar rats; ****p<0.001* vs. Intact female Wistar rats; ###

*p<0.001* vs. OVX ;+++*p<0.001* vs. OVX MI+EA by One-way ANOVA followed by Tukey’s *post hoc* test.
